# Influence of K_2_NbF_7_ Catalyst on the Desorption Behavior of LiAlH_4_

**DOI:** 10.3389/fchem.2020.00457

**Published:** 2020-06-12

**Authors:** Nurul Amirah Ali, Noratiqah Sazelee, Muhammad Syarifuddin Yahya, Mohammad Ismail

**Affiliations:** Energy Storage Research Group, Faculty of Ocean Engineering Technology and Informatics, Universiti Malaysia Terengganu, Terengganu, Malaysia

**Keywords:** hydrogen storage, lithium aluminum hydride, desorption, catalyst, metal halide

## Abstract

In this study, the modification of the desorption behavior of LiAlH_4_ by the addition of K_2_NbF_7_ was explored for the first time. The addition of K_2_NbF_7_ causes a notable improvement in the desorption behavior of LiAlH_4_. Upon the addition of 10 wt.% of K_2_NbF_7_, the desorption temperature of LiAlH_4_ was significantly lowered. The desorption temperature of the LiAlH_4_ + 10 wt.% K_2_NbF_7_ sample was lowered to 90°C (first-stage reaction) and 149°C (second-stage reaction). Enhancement of the desorption kinetics performance with the LiAlH_4_ + 10 wt.% K_2_NbF_7_ sample was substantiated, with the composite sample being able to desorb hydrogen 30 times faster than did pure LiAlH_4_. Furthermore, with the presence of 10 wt.% K_2_NbF_7_, the calculated activation energy values for the first two desorption stages were significantly reduced to 80 and 86 kJ/mol; 24 and 26 kJ/mol lower than the as-milled LiAlH_4_. After analysis of the X-ray diffraction result, it is believed that the *in situ* formation of NbF_4_, LiF, and K or K-containing phases that appeared during the heating process promoted the amelioration of the desorption behavior of LiAlH_4_ with the addition of K_2_NbF_7_.

## Introduction

The excessive consumption of fossil fuels and the emission of carbon dioxide are the roots of environmental pollution. As a resolution to this global issue, the utilization of clean, and sustainable energy resources such as hydrogen, wind, and solar has become an inescapable need. Recently, hydrogen has received a large amount of attention as a future energy carrier. Hydrogen promises to be a clean and renewable energy carrier. Moreover, the production of hydrogen can be achieved from various resources, both renewable (e.g., solar, wind, and hydro) and non-renewable (e.g., natural gas and coal; Winter, 2009; Parra et al., 2019). Furthermore, energy production via hydrogen-oxygen reaction will only produce water as a by-product (Crabtree et al., 2004).

In pursuit of the success of hydrogen as a future energy carrier, the need for an efficient and reliable storage method has become the top priority. In general, there are three forms of hydrogen storage which are: (i) compressed hydrogen gas, which requires high pressure, (ii) liquefaction, and (iii) solid-state hydrogen storage via hydrides (Dalebrook et al., 2013; Zhang et al., 2016; Barthelemy et al., 2017). Solid-state hydrogen storage has been perceived to be an efficient and favorable method because of its safety, storage requirements, and storage capacity.

Lithium aluminum hydride (LiAlH_4_) has major benefits and is the preferable solid-state material. LiAlH_4_ is attractive due to its low temperature of hydrogen release and high storage capacity (10.6 wt.%; Andrei et al., 2005; Ares et al., 2008). The desorption process of LiAlH_4_ occurs in three stages, as follows:

(1) $$\begin{array}{l} 3\text{LiAl}{\text{H}}_{4}\to \text{L}{\text{i}}_{3}\text{Al}{\text{H}}_{6}+2\text{Al}+3{\text{H}}_{2} \end{array}$$

(2) $$\begin{array}{l} \text{L}{\text{i}}_{3}\text{Al}{\text{H}}_{6}+2\text{Al}\to 3\text{LiH}+3\text{Al}+3/2{\text{H}}_{2} \end{array}$$

(3) $$\begin{array}{l} 3\text{LiH}+3\text{Al}\to 3\text{LiAl}+3/2\;{\text{H}}_{2} \end{array}$$

The first reaction (1) occurs in a temperature range of 150–175°C and desorbs 5.2 wt.% of the hydrogen. The second reaction (2) takes places at 180–220°C and desorbs 2.6 wt.% of the hydrogen, while the third reaction (3) happens at temperatures > 400°C, with 2.6 wt.% of the hydrogen desorbed.

In spite of its advantages, LiAlH_4_ has some shortcomings, such as irreversible and slow desorption kinetics (Pukazhselvan et al., 2012). Moreover, the thermal decomposition in reaction 3 is considered incompatible with applied applications due to its high requirement for temperature (>400°C) to release hydrogen. Tremendous efforts have been devoted to overcoming the shortcomings of LiAlH_4_, such as the implementation of the ball milling method (Balema et al., 2000, 2001; Liu et al., 2009) and impurity-doping with various catalysts such as metals (Resan et al., 2005; Xueping et al., 2009; Langmi et al., 2010; Varin and Parviz, 2012), metal oxides (Zhai et al., 2012; Li Z. et al., 2013; Li et al., 2014; Liu et al., 2014; Sulaiman and Ismail, 2017; Ali et al., 2019; Sazelee et al., 2019), Ti-based additives (Ismail et al., 2011; Amama et al., 2012; Wohlwend et al., 2012; Li L. et al., 2013), and metal halides (Fernandez et al., 2007; Suttisawat et al., 2007; Xueping et al., 2007; Sun et al., 2008; Li et al., 2012).

Among these catalysts, previous studies have revealed that metal halides provide essential catalytic effects on the performance of LiAlH_4_. Cao et al. (2018) reported that the addition of ScCl_3_ had a superior effect on the performance of lithium alanates. The desorption process of the LiAlH_4_-10 mol% ScCl_3_ sample began at a lower temperature (~120°C), while the undoped LiAlH_4_ released hydrogen from around 150°C. Besides, the time needed to complete the dehydrogenation process was shortened with the addition of 1–10 mol% ScCl_3_. Meanwhile, Sun et al. (2008) found that NiCl_2_ significantly boosted the desorption behavior of LiAlH_4_. A composite sample of LiAlH_4_-NiCl_2_ demonstrated three times the desorption rate of pure LiAlH_4_, which was not able to desorb any hydrogen at 100°C. It was believed that the LiAlH_4_-NiCl_2_ sample presented this notable improvement due to the formation of Ni, which plays a vital role in accelerating the LiAlH_4_-NiCl_2_ system. Another investigation of the catalytic effect of metal halides was carried out by Liu et al. (2012). They proved that a LiAlH_4_-TiCl_3_ sample could release hydrogen at a lower temperature (80°C) than the pure LiAlH_4_. Furthermore, the dehydrogenated sample had good cyclability, with the composite sample able to retain a high capacity for hydrogen (6.4 wt.%) even after completing the 3rd cycle. Moreover, Ismail et al. (2010) observed that the composite sample of LiAlH_4_-1 mol NbF_5_ showed a 5–6 times faster dehydrogenation rate than the milled LiAlH_4._ Additionally, the LiAlH_4_-NbF_5_ composite sample had lower activation energy; 67 kJ/mol (first-stage reaction) and 77 kJ/mol (second-stage reaction), respectively. However, the improvement of LiAlH_4_ through the addition of a catalyst is still lacking, and further enhancements still need to be carried out. Moreover, different catalysts will enable different effects and performances. Therefore, it is interesting to enhance the desorption performance of LiAlH_4_ by the addition of other metal halides.

Metal halides, especially fluorides, are known to be highly effective catalysts for solid-state materials (Sulaiman et al., 2016; Yap et al., 2017; Youn et al., 2017). A number of researchers have reported that niobium fluoride exhibits a notable effect on the hydrogenation behavior of metal hydrides and the complex hydrides. A study conducted by Luo et al. (2008) revealed that the addition of 2 mol of NbF_5_ led to faster desorption kinetics for the MgH_2_-NbF_5_ sample as compared to pure MgH_2_. At 573 K, the MgH_2_-NbF_5_ sample could desorb 4.7 wt.% of hydrogen, while the pristine MgH_2_ desorbed almost no hydrogen. Other than that, Kou et al. (2014) added NbF_5_ to LiBH_4_ and demonstrated notable improvement on the desorption performance of LiBH_4_. In comparison to the milled LiBH_4_, which started to desorbed hydrogen at >400°C, the composite sample of LiBH_4_-NbF_5_ had a lower desorption temperature, 60°C. Moreover, Wang and colleagues (Wang et al., 2020) showed that a composite of Mg(BH_4_)_2_-doped NbF_5_ possessed the best dehydrogenation performance, with the ability to release hydrogen at low temperature (120°C), as compared to amorphous Mg(BH_4_)_2_ (126.9°C), and pristine Mg(BH_4_)_2_ (282.7°C)_._ Meanwhile, Cheng et al. (2018) demonstrated that upon the addition of NbF_5_, the composite of 4LiBH_4_-MgH_2_-Al exhibited excellent kinetics and reversibility performance. It took <4 h to achieve 90% of the total amount of hydrogen desorption. Other than that, Xiao et al. (2012) proved that the performance of LiBH_4_/MgH_2_ was significantly improved with the addition of NbF_5_. Here, the addition of NbF_5_ not only had reduced the onset decomposition temperature but also improved the dehydrogenation and absorption rates.

On the other hand, potassium (*K*) is another well-known additive for hydrogen storage systems. Wang et al. (2009) demonstrated that the addition of *K* significantly boosted the desorption process of Mg(NH_2_)_2_/LiH by reducing the overall reaction temperature. Furthermore, Dong et al. (2014) revealed that superior results for the hydrogenation performance of the LiH-NH_3_ system were obtained by the addition of various potassium compounds.

In respect to this matter, it is interesting to mix niobium fluoride with potassium as a ternary compound in the form of K_2_NbF_7_ and to study its potential catalytic effect. To date, no studies have been conducted using doped K_2_NbF_7_ as a catalyst for LiAlH_4._ Moreover, previous studies reported that K_2_NbF_7_ enables a remarkable improvement in the hydrogen storage performance of MgH_2_ (Yahya et al., 2018; Yahya M. S. and Ismail M., 2018). Thus, it is of great interest to explore the influence of K_2_NbF_7_ on the desorption performances of LiAlH_4_. It is anticipated that the addition of K_2_NbF_7_ will have notable effects on the desorption and kinetic performances of LiAlH_4_.

## Experimental Details

Commercial powders of LiAlH_4_ (purity 95%) and K_2_NbF_7_ (purity 98%) were obtained from Sigma Aldrich and were used without any modification. To minimize exposure to oxygen and water moisture, the samples were prepared and handled in the Ar-filled Mbraun Unilab glove box. In this study, 10 wt.% of K_2_NbF_7_ was mechanically milled together with LiAlH_4_ to explore its effect on the desorption behavior of LiAlH_4_. The milling process was done in a planetary ball mill (NQM-0.4) for 1 h, starting with 0.5 h of milling, followed by 6 min of rest time, and then another 0.5 h of milling in a different rotation direction at a speed of 400 rpm. The samples were placed in a hardened stainless-steel jar with four stainless balls, each 1 cm in size. The ratio of the balls to the weight of the powder was 40:1. For comparison purposes, the as-received LiAlH_4_ was treated under the same conditions.

The hydrogenation performances of LiAlH_4_ + 10 wt.% K_2_NbF_7_ were studied with temperature-programmed desorption (TPD) using Sievert-type pressure-composition-temperature (PCT) equipment (Advanced Materials Corporation). In order to determine the initial decomposition temperature, the sample was heated from room temperature to 250°C (heating rate: 5°C/min). Other than that, the desorption kinetics performances were evaluated at 90°C under 1.0 atm of pressure. The apparent activation energy, *E*_*A*_, was determined using differential scanning calorimetry (DSC, Mettler Toledo, DSC/TGA 1), loading 5–7 mg of the samples into a crucible and heating from 25 to 300°C at heating ramps of 15, 20, 25, and 30°C/min under an argon flow (50 ml/min). In terms of the morphology and phase structure characterizations, the samples were analyzed using scanning electron microscopy (SEM: JEOL JSM 6350LA), X-ray diffractometry (XRD, Rigaku Miniflex), and Fourier transform infrared (IR Shimadzu Tracer-100).

## Results and Discussion

**Figure 1:**
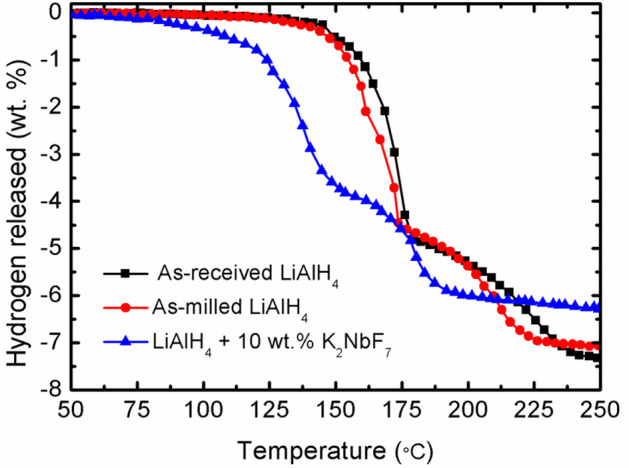
TPD profile for the as-received LiAlH_4_, as-milled LiAlH_4_, and LiAlH_4_ + 10 wt.% K_2_NbF_7_.

Figure 1 demonstrates the TPD results of the LiAlH_4_ and modified LiAlH_4_ system. The results show that the as-received and as-milled LiAlH_4_ have similar desorption processes that occur in two stages of desorption, as in Equations (1, 2), with 7.4 wt.% hydrogen capacity. Before the ball milling process, the first stage of desorption occurred at 147°C, with 5 wt.% of hydrogen released. Meanwhile, the desorption process for the second stage was recorded to happen at around 175°C, with a capacity of 2.4 wt.% of the hydrogen. After the milling process, the initial desorption temperature of the sample was similar to that of pure LiAlH_4_ but with slight temperature reductions to 144°C (first stage) and 174°C (second stage). This phenomenon showed that the 1-h milling process had an insignificant effect on the desorption behavior of LiAlH_4_. In contrast, the addition of 10 wt.% of K_2_NbF_7_ significantly decreased the decomposition temperature for both stages, to 90 and 149°C. However, the amount of hydrogen released from the LiAlH_4_ + 10 wt.% K_2_NbF_7_ sample was decreased to 6.3 wt.%. This is expected due to the dead weight of K_2_NbF_7_, which does not hold any hydrogen.

**Figure 2:**
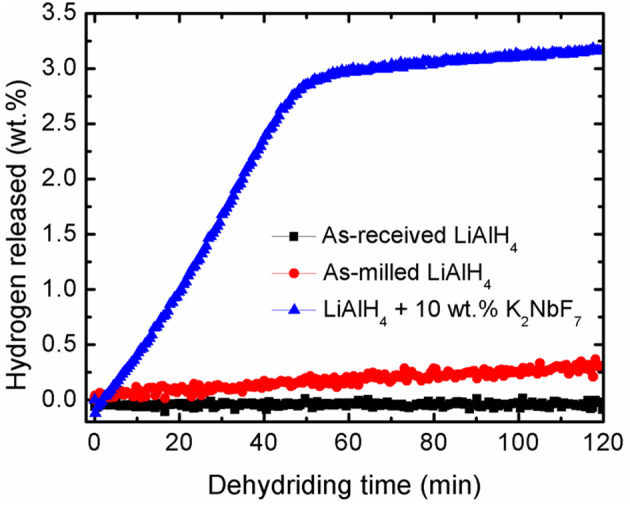
Dehydriding kinetics curves of as-received LiAlH_4_, as-milled LiAlH_4_, and LiAlH_4_ + 10 wt.% K_2_NbF_7_ at 90°C.

Further study on the catalytic activity of K_2_NbF_7_ was performed based on the desorption kinetics experiment. Figure 2 depicts a comparison of the hydrogen desorption at 90°C for LiAlH_4_ and LiAlH_4_ modified by the addition of 10 wt.% K_2_NbF_7_. It is noticeable that within 120 min, the undoped LiAlH_4_ was only able to desorb a small amount of hydrogen; 0.1 wt.% for the as-received LiAlH_4_ and 0.4 wt.% for the as-milled LiAlH_4._ Surprisingly, with the addition of 10 wt.% K_2_NbF_7_, the doped sample desorbed ~3.2 wt.% H_2_ within the same duration. This desorption rate was 30 times faster than that of the as-received LiAlH_4_. This enhancement may be correlated to the formation of surface defects and active materials through the reaction of the LiAlH_4_ + 10 wt.% K_2_NbF_7_ composite (Cai et al., 2016).

**Figure 3:**
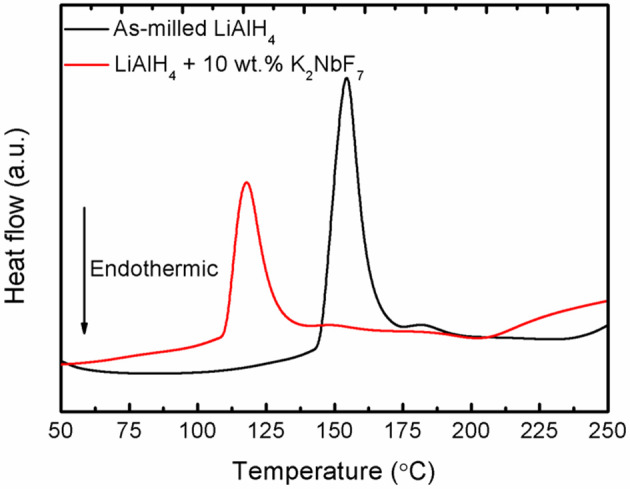
DSC traces of the as-milled LiAlH_4_ and LiAlH_4_ + 10 wt.% K_2_NbF_7_ (heating ramp: 15°C/min).

In terms of thermal behavior, DSC experiments were conducted for the doped and un-doped LiAlH_4_ samples. Figure 3 displays the DSC curves of the samples at a heating ramp of 15°C/min. Both the doped and un-doped LiAlH_4_ have two endothermic and exothermic peaks. The first exothermic peak corresponds to the reaction of LiAlH_4_ with surface hydroxyl groups, while the first endothermic peak is ascribed as its melting process. The second exothermic peak is attributed to the decomposition of LiAlH_4_, as described in Equation (1), and the second endothermic peak correlates with the decomposition of Li_3_AlH_6_, as described by Equation (2). Both samples exhibit similar thermal behavior, but the peaks of the LiAlH_4_ + 10 wt.% K_2_NbF_7_ sample occur at a lower temperature as compared to as-milled LiAlH_4_.

**Figure 4:**
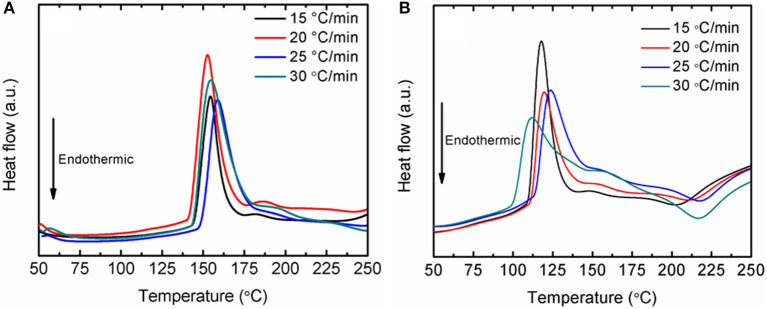
DSC traces at various heating ramps of the LiAlH_4_ when **(A)** as-milled and **(B)** with 10 wt.% of K_2_NbF_7_.

**Figure 5:**
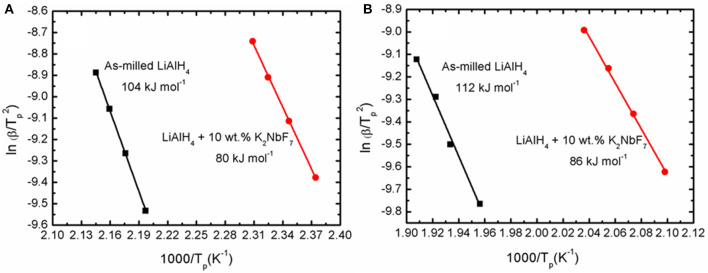
Corresponding Kissinger plots of the as-milled LiAlH_4_ and LiAlH_4_ + 10 wt.% K_2_NbF_7_ for the **(A)** first stage and **(B)** second stage of reaction.

Fundamentally, the enhancement of the initial temperature to release hydrogen and the faster desorption kinetics rates are correlated with the energy barrier of LiAlH_4_. In this study, the decomposition activation energy (*E*_*A*_) is the least possible amount of energy needed by LiAlH_4_ to begin the hydrogen desorption process. Figure 4 shows DSC traces for several heating ramps (15, 20, 25, and 25°C/min). By referring to the plots, the activation energies for both decomposition stages of the as-milled LiAlH_4_ and LiAlH_4_ + 10 wt.% K_2_NbF_7_ samples were determined using the Kissinger analysis, as in equation (4):

(4) $$\begin{array}{l} \ln\;\left[\beta /{T_{p}}^{2}\right]=-E_{A}/RT_{p}+A \end{array}$$

where β, *T*_*p*_, *R*, and *A* are the heating rate, peak temperature in the DSC curve, gas constant, and linear constant, respectively. The apparent activation energy was determined from the slope of ln [β / *T*_*p*_^2^] versus 1000/*T*_*p*_, as shown in Figure 5.

The activation energy was calculated based on the second exothermic (decomposition of LiAlH_4_) and second endothermic (decomposition of Li_3_AlH_6_) reactions. For the as-milled LiAlH_4_, the activation energy values were 104 and 112 kJ/mol for the first two stages of reaction, respectively. After the addition of 10 wt.% K_2_NbF_7_, the activation energy values dropped to 80 kJ/mol (first stage) and 86 kJ/mol (second stage), 23% lower than those of the un-doped LiAlH_4_. These results are in good agreement with other studies that prove the addition of a catalyst is able to reduce the activation energy of LiAlH_4_. Table 1 lists the activation energy from previous studies for comparison purposes. The reduction in these activation energies verifies that K_2_NbF_7_ plays a major role in enhancing the desorption kinetics performance of LiAlH_4._

**Table 1:** Activation energy of catalyst-doped LiAlH_4_ from previous studies.

**System**	**Activation energy (kJ/mol)**		**References**
	**First stage**	**Second stage**	
LiAlH_4_+K_2_TiF_6_	78.20	90.80	Li et al., 2012
LiAlH_4_+Ti_3_C_2_	79.81	99.68	Xia et al., 2019
LiAlH_4_+FeCl_2_	81.48	105.01	Cai et al., 2016
LiAlH4+ScCl_3_	82.30	93.20	Cao et al., 2018
LiAlH_4_+Co@C	95.36	115.60	Li et al., 2015

**Figure 6:**
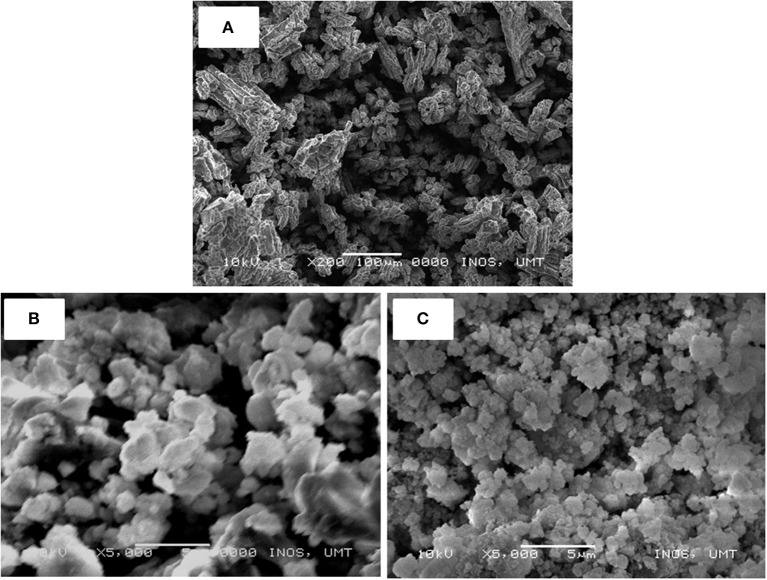
SEM images of LiAlH_4_ when **(A)** as-received, **(B)** as-milled, and **(C)** with 10 wt.% of K_2_NbF_7_.

The morphological structures of the doped and un-doped LiAlH_4_ were examined using SEM equipment. Figure 6 shows SEM images of the un-doped and doped-LiAlH_4_ samples. As shown in Figure 6, the pure LiAlH_4_ exhibits larger particle sizes than the milled sample. The as-received LiAlH_4_ (Figure 6A) has larger (15–40 μm), non-uniform rod-shaped particles. Furthermore, the as-received LiAlH_4_ shows a uniform size distribution and consists of “blocky” particles, consistent with the report by Varin and Zbroniec (2010). Meanwhile, after 1 hour of milling, the milled LiAlH_4_ (Figure 6B) displays a reduction in particle sizes but with some agglomeration and inconsistency in particle size. Then, with the addition of 10 wt.% of K_2_NbF_7_ (Figure 6C), the morphological structure of the sample was notably enhanced. The doped sample has smaller particle sizes and is less agglomerated. This observation is in line with numerous research results that have shown a reduction of particle sizes with the addition of a catalyst (Aguey-Zinsou et al., 2007; Ali et al., 2018; Yahya M. and and Ismail M., 2018). In this study, K_2_NbF_7_ functioned as a dispersing agent that impeded the sample from agglomerating. The particle size is important because smaller particles provide more area for surface defects and additional grain boundaries (Schulz et al., 1999; Sakintuna et al., 2007; Ranjbar et al., 2009). As a consequence, the desorption kinetics of LiAlH_4_ will be improved.

**Figure 7:**
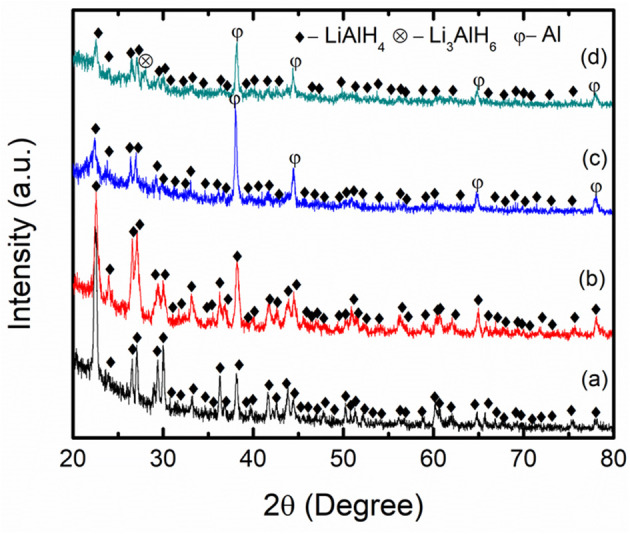
XRD patterns of **(a)** as-received LiAlH_4_, **(b)** as-milled LiAlH_4_, **(c)** LiAlH_4_ + 10 wt.% K_2_NbF_7_, and **(d)** LiAlH_4_ + 30 wt.% K_2_NbF_7_.

Figure 7 presents the XRD profiles of the as-received LiAlH_4_, as-milled LiAlH_4_, and LiAlH_4_-K_2_NbF_7_ sample. The XRD characterization was performed to explore the reaction process and the mechanism that operated during the milling process. Figure 7A displays the XRD pattern of the as-received LiAlH_4_ and shows that only the LiAlH_4_ phase was detected, which confirms the purity of the LiAlH_4_. The XRD pattern of the milled LiAlH_4_ Figure 7B shows similar peaks to the as-received LiAlH_4_. This result shows that LiAlH_4_ has high stability during the milling process and agrees well with a previous study (Ismail et al., 2010). Meanwhile, with the addition of 10 wt.% of K_2_NbF_7_ (Figure 7C), only LiAlH_4_ and Al peaks are visible and no peak of K_2_NbF_7_ was detected, suggesting that the amount of catalyst was too small to be picked up by the XRD. The appearance of Al peaks indicates that a part of the LiAlH_4_ had decomposed to Li_3_AlH_6_ and Al (reaction 1) during the milling process in the presence of 10 wt.% K_2_NbF_7_. Surprisingly, the XRD result for the 10 wt.% K_2_NbF_7_-doped LiAlH_4_ sample does not show any peaks of Li_3_AlH_6_. Additional characterization was carried out for a doped sample with 30 wt.% K_2_NbF_7_ (Figure 7D). A K_2_NbF_7_ peak was against not detected by the XRD for this sample. This may be because the K_2_NbF_7_ is in an amorphous state. Similar phenomena were reported by previous studies, where several catalysts like TiO_2_ and TiF_3_ were not detected by the XRD after the milling process (Ismail et al., 2011; Zang et al., 2015). However, for the LiAlH_4_ + 30 wt.% K_2_NbF_7_ sample, diffraction peaks corresponding to the decomposition product, Al and Li_3_AlH_6_, were detected. Meanwhile, unlike for the LiAlH_4_ + 10 wt.% K_2_NbF_7_ sample, for which only peaks of Al were detected while peaks of Li_3_AlH_6_ could not be discovered by XRD.

**Figure 8:**
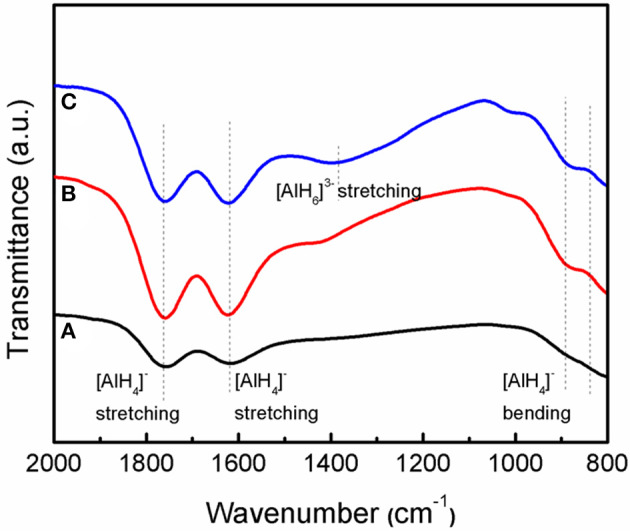
IR spectra of the LiAlH_4_ when **(A)** as-received, **(B)** as-milled, and **(C)** with 10 wt.% of K_2_NbF_7_.

Figure 8 shows the IR spectra of the as-received LiAlH_4_, as-milled LiAlH_4_, and LiAlH_4_ + 10 wt.% K_2_NbF_7_ in the range of 800 to 2,000 cm^−1^. The FTIR characterizations were conducted to identify the presence of Li_3_AlH_6_ in the 10 wt.% K_2_NbF_7_-doped LiAlH_4_ sample. For all samples, two distinct regions of Al-H modes were detected at around 800–900 cm^−1^ ([AlH_4_]^−^ stretching modes) and 1,600–1,800 cm^−1^ ([AlH_4_]^−^ bending modes), respectively. Furthermore, with the addition of 10 wt.% K_2_NbF_7_, a weak IR absorption peak at 1,398 cm^−1^ was detected, which indicates the presence of Li_3_AlH_6_. This result suggests that with the addition of 10 wt.% K_2_NbF_7_, LiAlH_4_ was partially decomposed to Li_3_AlH_6_ and Al (reaction 1) during the milling process, consistent with the XRD results (Figure 7C).

**Figure 9:**
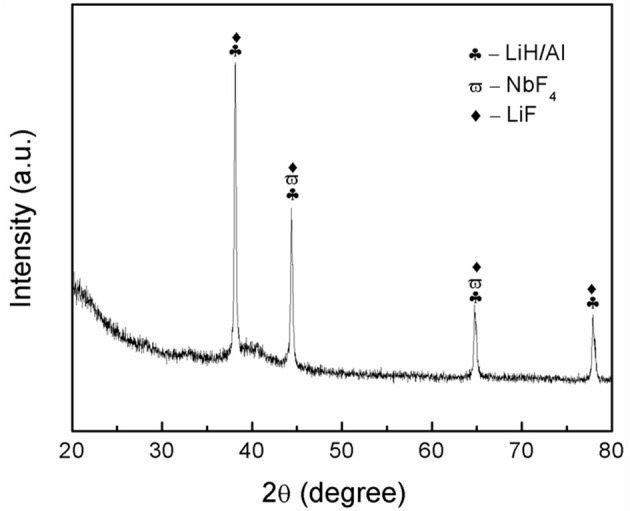
XRD pattern of dehydrogenated LiAlH_4_ + 10 wt.% K_2_NbF_7_ sample at 250°C.

To investigate the specific mechanism that is related to the enhanced desorption performance of LiAlH_4_, the dehydrogenated sample was examined using XRD. The XRD pattern for the dehydrogenated sample is depicted in Figure 9. After the dehydrogenation process at 250°C, the main peaks observed are the LiAlH_4_ dehydrogenation products, LiH and Al, which indicates complete dehydrogenation of LiAlH_4_. In addition, peaks for LiF and NbF_4_ were detected after the dehydrogenation process. However, the peak of the K-containing phase was not detected after the dehydrogenation process, potentially due to the low amount of catalyst.

Niobium fluoride is well established as a promising catalyst that plays a vital role in enhancing the hydrogenation performance of solid-state material (Luo et al., 2007; Malka et al., 2011; Mao et al., 2013). It is reasonable to state that the NbF_4_ that formed *in situ* after the desorption process contributes to a remarkable amelioration of the desorption behavior of LiAlH_4_. This result well-agreed with previous research that demonstrates the outstanding dehydrogenation performance of LiAlH_4_-NbF_5_ (Ismail et al., 2010). On the other hand, the LiF formed was believed to significantly affect the hydrogenation behavior of the doped sample based on work carried out as by Gosalawit-Utke et al. (2010). Additionally, it is believed that the formation of LiF plays a similar role in the growth of LiH and Al, since LiF has a similar cubic structure (space group: Fm-3m; Y. Liu et al., 2010). Also, LiF crystallites act as nucleation sites and facilitate the growth of LiH and Al crystallites, which promotes to the change of the nucleation morphology. These two factors significantly contribute to the kinetics enhancement achieved in the doped sample. Additionally, it is believed that *K* or K-containing phases also play a vital role in enhancing the desorption behavior of LiAlH_4_. This was deduced based on successful previous work on the application of K as a catalyst for solid-state materials (Wang et al., 2009; Dong et al., 2014). Therefore, it can be concluded that the *in situ* formation of LiF, NbF_4_, and K or K-containing phases synergistically contributed to the amelioration of the dehydrogenation kinetics of LiAlH_4_.

## Conclusion

K_2_NbF_7_ demonstrated an excellent catalytic effect on the desorption behavior of LiAlH_4_. The initial temperatures at which LiAlH_4_ + 10 wt.% K_2_NbF_7_ released hydrogen, 90 and 149°C for the first two stages, were lower than those of the as-milled LiAlH_4_ (147 and 175°C). In terms of desorption kinetics behavior, the LiAlH_4_ + 10 wt.% K_2_NbF_7_ released 3.2 wt.% of hydrogen within 120 min, which is 30 faster than the pure LiAlH_4_. The addition of K_2_NbF_7_ significantly reduced the decomposition activation energy from 104 to 80 kJ/mol for the first stage and 112 to 86 kJ/mol for the second stage. The XRD spectra suggested that the *in situ* formation of LiF, NbF_4_, and K or K-containing phases acted as boosters and ameliorated the dehydrogenation behavior of LiAlH_4_. This work demonstrates that K_2_NbF_7_ was has a superior catalytic effect and confers better desorption behavior to LiAlH_4_.

## Data Availability Statement

The datasets generated for this study are available on request to the corresponding author.

## Author Contributions

All authors listed have made a substantial, direct and intellectual contribution to the work, and approved it for publication.

## Conflict of Interest

The authors declare that the research was conducted in the absence of any commercial or financial relationships that could be construed as a potential conflict of interest.
